# Results

**Published:** 1903-02

**Authors:** 


RESULTS.—It is very gratifying to note that a widespread popular sentiment in favor of sanitary legislation has been awakened, and the people are beginning to demand it. The 7500 pamphlets, "In the Interest of the Public Health; a Plea for Preventive Measures," sent out by the State Medical Association committee, seem to have done a world of good. The committee reserved about 300 copies at the time the pamphlet was mailed (a copy to every lawyer in Texas and to every member of the Legislature), and they have since the meeting of the Legislature been put into the hands of the members. Some of the members have been given several copies, which they have mailed.

The "Red Back" claims no little of the credit for this awakening of the public sentiment. It has preached sanitation in every issue nearly for eighteen years.

One of the signs of the awakening is the introduction in the Senate by Senator Harper of a bill (separate from the committee's bill, which really provides for the same thing) to authorize the State Health Officer to prescribe rules and regulations and enforce them under penalty for the disinfection of all railroad cars and other public conveyances, and the introduction in both houses of bills for a State hospital for indigent consumptives.

A reform is on, and it will be far reaching for good. Let every reputable doctor now put his shoulder to the wheel and let us make "a long pull, a strong pull and a pull all together."

---

THE STATE BOARD OF MEDICAL EXAMINERS will ask the Legislature to amend the medical practice act as follows:

Section 4. Amend by adding to the last line, after the words "surgery and midwifery" the following: "The said boards shall each procure a seal, which shall be the official seal of the boards."

Section 5. Amend by striking out the words "seal of the State of Texas" in the last line and adding instead "the seal of the board granting such license."

Section 6. Amend by adding in line twenty-two, after the word "character," "and that he or she is a graduate of a reputable medical school of recognized standing, requiring for graduation attendance upon not less than three regular sessions, of not less than six months each, in three separate calendar years."

Section 8. Amend by adding in line twenty-five after the words "receive a license from the Board of Medical Examiners of Texas to practice in this State" the following: "Provided, that the medical laws and examining boards of such States and Territories grant equal rights and recognition to the licentiates of the boards herein created."

Section 13. Amend by adding in the fourth line, after the word "imprisonment," the following: "In the county jail." And by striking out in the seventh line, after the words "surgeon or midwife," the following sentence: "Provided, that the provisions of this act do not apply to persons treating disease who do not prescribe or give drugs or medicine."

There is another amendment worse needed than the above. It is this: The board should have the power to refuse to license a doctor, no matter how many diplomas he may have nor how good an examination he may stand, who puts out such a lying decoy as the following, which I take from the *Austin Daily Statesman*. No name is attached to this advertisement, and but one person is in attendance at the "British Medical Institute." I learn that he is a young man—a recent graduate of the Marion Sims Medical College, and that he is applying for license—and has been given an examination by Dr. Smith, Secretary Board of Medical Examiners. I do not know with what result. The dodge is that no charge is made. The law should be amended to meet this. He is "practicing medicine" and presumably selling medicines. I understand that the case will be looked into:

"THE DOCTORS ARE HERE.

"THREE MONTHS' SERVICES ARE GIVEN FREE TO ALL INVALIDS WHO CALL BEFORE FEBRUARY 17.

"A staff of eminent physicians and surgeons from the British Medical Institute have, at the urgent solicitation of a large number of patients under their care in this country, established a permanent branch of the institute in this city in the Sampson building, rooms 8 and 9.

"These eminent gentlemen have decided to give their services entirely free for three months (medicines excepted) to all invalids who call upon them for treatment between now and February 17. These services consist not only of consultation, examination and advice, but also of all minor surgical operations.

"The object in pursuing this course is to become rapidly and personally acquainted with the sick and afflicted, and under no conditions will any charge whatever be made for any services rendered for three months to all who call before February 17.

"The doctors treat all forms of disease and deformities, and guarantee a cure in every case they undertake. At the first interview a thorough examination is made, and if incurable, you are frankly and kindly told so; also advised against spending your money for useless treatment.

"Male and female weakness, catarrh and catarrhal deafness, also varicocele, rupture, goitre, cancer, the opium habit, skin diseases and all diseases of the rectum are positively cured by their new treatment.

"The chief associate surgeon of the institute is in personal charge.

"Office hours from 9 a. m. till 8 p. m.

"No Sunday hours.

"SPECIAL NOTICE.—If you can not call, send stamp for question blank for home treatment."

---

The supreme court of Illinois and of Alabama have decided that osteopaths, although they "give no medicine," are practicing medicine within the meaning of the statute, and are amenable to the law for its violation. Our practice act should be amended so as to bring these fellows and all other "drugless terrors" before the examining board.

---

THE "BRITISH MEDICAL INSTITUTE" and the "staff of eminent British doctors in charge," as per the flaming advertisement in the *Austin Daily Statesman,* reproduced elsewhere in this issue, came to grief after said article was in type. The*Austin Daily Tribune* gives the following account of it—up to the time the trial in the justice's court was on:

"A TRAVELING MEDICAL MAN HAS TROUBLE.

"A Local Physician of the British Medical Institute Arrested for Practicing Medicine Without a License.

"HE HAS NO STATE LICENSE TO PRACTICE.

"State Law Violated and the Alleged Doctor is on Trial this Afternoon Before Justice White.

"Arrested on the charge of practicing medicine without a license, Dr. Dearduff of the local branch of the British Medical Institute, is now standing trial before Justice White. The complainants are Dr. F. E. Daniel, President of the Austin District Medical Society, and Dr. T. J. Bennett. Yesterday evening they appeared before Justice White and swore out affidavit charging Dr. Dearduff with violating the State medical law, which provides that before being eligible to practice medicine, obstetrics or surgery in Texas a physician must stand examination before the State Board of Medical Examiners. This, it is claimed, Dr. Dearduff has failed to do.

"The law dates back to 1901 and was passed in order to protect the public from quacks and imposters, and create confidence that none but duly qualified physicians should practice medicine.

"The advertisements of the British Medical Institute claim that they treat diseases free, but evidence has been adduced to show that such is not so, inasmuch as a receipt was issued to a man for $5 for a half month's treatment. In any event, the law may be construed to include within its meaning any and all persons who practice medicine, whether they give their services and drugs or charge for them.

"The punishment for the offense is a fine of not less than $50 nor more than $500, or imprisonment for six months or both.

"The case went on trial at 2 o'clock and is not yet concluded. (At hour of *Tribune* going to press.)

It is hardly necessary to add that the one-man-staff of eminent

British doctors was held under $200 bond for trial at April term of county court. The case is a clear one, and the evidence of violation of the law is conclusive. There is no doubt of a conviction if he lets it come to trial. It is thought he will plead guilty, pay the fine and get out. Look out for him all you fellows who are interested in the enforcement of the medical practice act, and the suppression of quackery.

